# Knowledge Update on the Economic Evaluation of Pacemaker Telemonitoring Systems

**DOI:** 10.3390/ijerph182212120

**Published:** 2021-11-18

**Authors:** Antonio Lopez-Villegas, César Leal-Costa, Mercedes Perez-Heredia, Irene Villegas-Tripiana, Daniel Catalán-Matamoros

**Affiliations:** 1 Social Involvement of Critical and Emergency Medicine, CTS-609 Research Group, Poniente Hospital, 04700 El Ejido-Almería, Spain; antoniolopezvillegas@andaluciajunta.es; 2 Nursing Department, University of Murcia, 30120 Murcia, Spain; 3 Research Management Department, Primary Care District Poniente of Almería, 04700 El Ejido-Almería, Spain; mercedes.perez.heredia.sspa@juntadeandalucia.es; 4 Research Support Unit and Library, Poniente Hospital, 04700 El Ejido-Almería, Spain; irene.villegas@ephpo.es; 5 UC3M MediaLab, Department of Communication and Media Studies, Madrid University Carlos III, 28903 Madrid, Spain; dacatala@hum.uc3m.es; 6 Health Sciences Research Institute, University of Almería, 04120 Almería, Spain

**Keywords:** cost-benefit analysis, follow-up studies, health-related quality of life, pacemakers, remote telemonitoring, telemedicine

## Abstract

(1) Introduction: In the last two decades, telemedicine has been increasingly applied to telemonitoring (TM) of patients with pacemakers; however, presently, its growth has significantly accelerated because of the COVID-19 pandemic, which has pushed patients and healthcare workers alike to seek new ways to stay healthy with minimal physical contact. Therefore, the main objective of this study was to update the current knowledge on the differences in the medium-and long-term effectiveness of TM and conventional monitoring (CM) in relation to costs and health outcomes. (2) Methods: Three databases and one scientific registry were searched (PubMed, EMBASE, Scopus, and Google Scholar), with no restrictions on language or year of publication. Studies published until July 2021 were included. The inclusion criteria were: (a) experimental or observational design, (b) complete economic evaluation, (c) patients with implanted pacemakers, and (d) comparison of TM with CM. Measurements of study characteristics (author, study duration, sample size, age, sex, major indication for implantation, and pacemaker used), analysis, significant results of the variables (analysis performed, primary endpoints, secondary endpoints, health outcomes, and cost outcomes), and further miscellaneous measurements (methodological quality, variables coded, instrument development, coder training, and intercoder reliability, etc.) were included. (3) Results: 11 studies met the inclusion criteria, consisting of 3372 enrolled patients; 1773 (52.58%) of them were part of randomized clinical trials. The mean age was 72 years, and the atrioventricular block was established as the main indication for device implantation. TM was significantly effective in detecting the presence or absence of pacemaker problems, leading to a reduction in the number of unscheduled hospital visits (8.34–55.55%). The cost of TM was up to 87% lower than that of CM. There were no significant differences in health-related quality of life (HRQoL) and the number of cardiovascular events. (4) Conclusions: Most of the studies included in this systematic review confirm that in the TM group of patients with pacemakers, cardiovascular events are detected and treated earlier, and the number of unscheduled visits to the hospital is significantly reduced, without affecting the HRQoL of patients. In addition, with TM modality, both formal and informal costs are significantly reduced in the medium and long term.

## 1. Introduction

Telemedicine is the delivery of healthcare services with the help of information and telecommunication technology. Because of the enormous progress made in these technological fields, more and more hospitals have adopted electronic health records, leading to an exponential growth in the use of telemedicine [1,2]. Conventionally, telemedicine has been used to encourage self-care through remote and chronic disease monitoring, to provide consultations to patients who are unable to attend in-person (face-to-face) appointments, and to improve patient care within hospitals and clinics. A key advantage of telemedicine is its ability to increase access to health care by offering patients the opportunity to receive care in their homes and communities [2,3,4]. This becomes more important in the present COVID-19 pandemic, as both patients and health care workers are adopting methods with minimal physical contact [2].

Cardiovascular diseases affect heart and blood vessels and are the leading cause of death globally. A pacemaker is a device that is widely used in cardiac patients to restore normal heart rates, and patients with implanted pacemakers must be followed up regularly. Telemonitoring (TM) systems or remote monitoring of pacemakers provide a convenient means for regular assessment of device-related parameters, such as lead impedance and battery status, which may allow early detection of device and lead malfunctions [5,6,7,8,9,10]. Based on this, if required, changes in medication can be prescribed without consuming time and medical resources [5,11,12,13,14,15] and causing discomfort to patients and their caregivers [16]. Research indicates that clinically actionable events are detected sooner with telemonitoring than with standard in-office follow-ups [5,16], thereby allowing clinicians to act on these issues before they cause increased morbidity, hospitalizations, and costs [5]. Several studies have shown that TM represents an effective and cost-saving way in which to significantly reduce in-office follow-up visits and lower the burden for both hospitals and patients and their caregivers [17,18,19,20,21,22,23,24,25,26,27,28] without compromising safety [5,28,29,30]. Besides, TM has been associated with high patient acceptance, satisfaction, and quality of life as it entails less travel time, time off work, and interruption of patient activities, as well as increased adherence to programmed follow-up [5,31,32,33]. However, in spite of this, TM of users with pacemakers has not been universally adopted [34,35,36] and even hospitals that have incorporated this technology into routine clinical practice for other Cardiac Implantable Electronic Devices (CIEDs) do not routinely use it for pacemakers [37].

The recent strong growth in the number of patients with remotely monitored pacemakers has generated the need for studies comparing TM to conventional in-hospital monitoring (CM). Therefore, the main objective of this study was to conduct a systematic review analyzing the current scientific literature to evaluate the effectiveness and costs of both monitoring modalities

## 2. Methods

This systematic review has followed PRISMA guidelines, and the study has been registered in PROSPERO (PROVISIONAL ID number: 290,328).

### 2.1. Search Strategy

A structured review of the following databases was conducted: Medline via PubMed, EMBASE, Scopus, and Google Scholar. The Boolean operators used were AND OR. The following English search terms were used: “Pacemaker”, “Telemedicine”, and “Cost-Benefit Analysis.” These terms were searched in all the selected databases and in complete articles, including the title, summary, text, and keywords. The inclusion criteria for studies were (a) experimental or observational design; (b) studies based on complete economic evaluations, i.e., studies comparing health outcomes and costs, with no exclusions for analysis method (cost-effectiveness, cost-utility, cost-benefit, and cost-minimization); (c) patients with pacemakers, and (d) comparison of TM with CM. The search was conducted between 13 and 21 July 2021, with no restrictions on language or year of publication. In addition to the above-mentioned databases and registers, bibliographic references of interest, including systematic reviews and meta-analyses, were hand-searched.

### 2.2. Data Extraction

**Figure 1:**
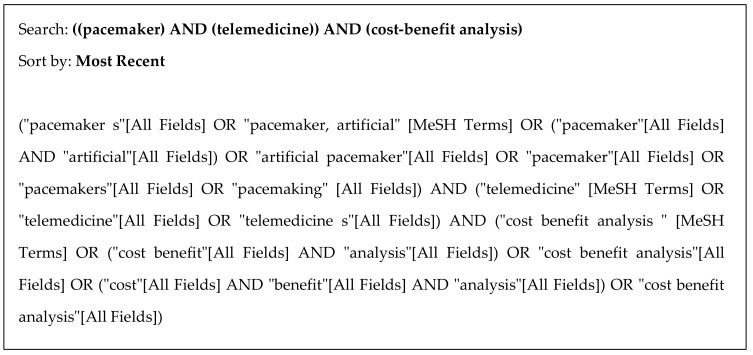
Search strategy used in MEDLINE (via PubMed).

The extraction and reading of all the titles and abstracts of the selected studies (Figure 1) were carried out independently by two researchers (M.P.-H. and I.V.-T.) in the first week of August 2021. As stated in the study aims, articles that could potentially meet the inclusion criteria were preselected. In the second half of August, the same two investigators read the full texts of the previously screened articles. In case of any disagreement between the two investigators regarding the inclusion or exclusion of an article, a third investigator (D.C.-M.) mediated. The variables included in the data analysis were (a) study characteristics (author, year of publication, country, study duration, sample size, age, sex, main indication for implantation, and pacemaker used) and (b) analysis and significant results of the variables (analysis performed, primary endpoints, secondary endpoints, health outcomes, and cost outcomes). Two researchers (C.L.-C. and D.C.-M.) independently evaluated the methodological quality of the selected articles using the checklist of López-Bastida et al. [38] as an assessment tool.

### 2.3. Variables Coded, Instrument Development, Coder Training, and Intercoder Reliability

Two researchers (A.L.-V. and M.P.-H.) tested the initial draft of the coding instrument informally by independently coding 10 print papers from the list of 1438 studies initially screened (title, abstract, and text), 6 articles from PubMed, and 4 from Google Scholar. Based on this, any issues and disagreements related to coding were discussed, and the form was revised. This protocol was repeated three times until the instrument was considered reliable, then a reliability pilot test was formally conducted using the below-mentioned methods. To establish the intercoder reliability, both researchers coded 88 (70.97%) papers from the list of 124 full-text revised studies. In addition, each researcher coded half of the remaining 36 articles. To build the final database, the papers used in the reliability analysis were divided randomly into two different groups, and the decisions of each coder were randomly selected. To assess intercoder reliability for each variable, percent agreement, Scott’s pi, Cohen’s kappa, and Krippendorff’s alpha were utilized [39], and ReCal (“Reliability Calculator”) software was used to calculate these variables [40,41]. In the case of two coders evaluating the same variable, Holsti’s method was not included because it is identical to Scott’s pi. Besides, to consider the coding of a variable reliable, either a Krippendorff’s alpha of ≥ 0.70 or a percent agreement of ≥ 0.90 is needed.

## 3. Results

**Figure 2:**
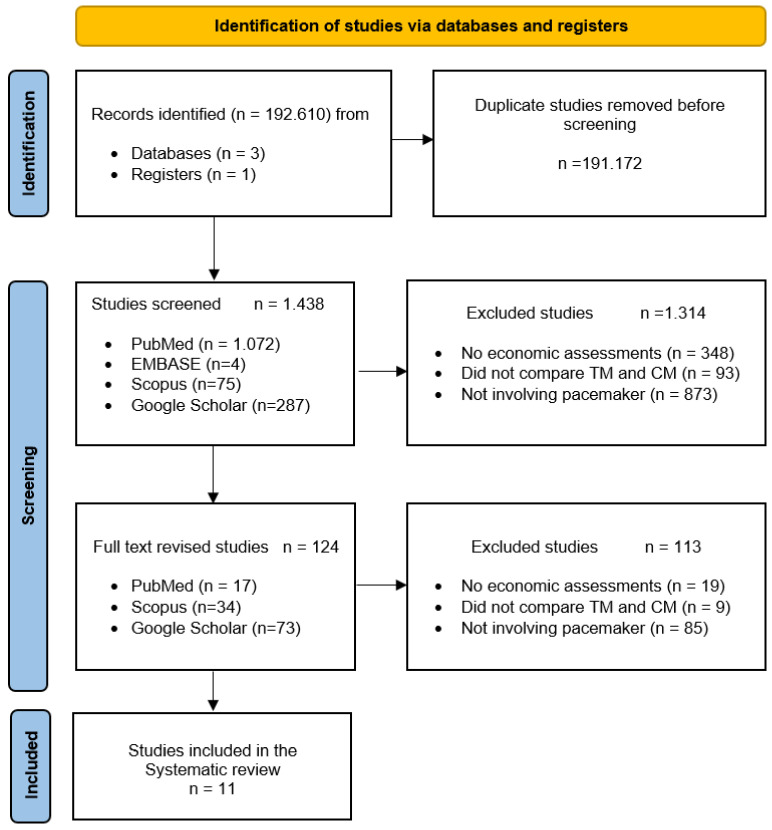
PRISMA flow diagram [52] of the selection process of studies for the systematic review of economic evaluations of remote monitoring systems and follow-up of patients with pacemakers. CM—Conventional monitoring; TM—Telemonitoring.

The literature search identified 1438 articles. After the first screening, the full texts of 124 relevant studies were reviewed. Out of these 11 articles [7,42,43,44,45,46,47,48,49,50,51], corresponding to 10 different studies (references [49,51] belong to the same study), met the selection criteria (Figure 2) and were included in the subsequent synthesis of evidence. The references from the 113 excluded articles are available in the Supplementary Material (File S1). Because of the substantial heterogeneity of the selected manuscripts, a meta-analysis could not be carried out.

### 3.1. Characteristics of the Selected Studies

This review included seven experimental [7,42,47,48,49,50,51] and four descriptive/observational [43,44,45,46] studies and aimed to evaluate the results on quality of life, effectiveness, safety, reliability, and costs of TM of pacemakers compared with CM [7,42,43,44,45,46,47,48,49,50,51].

The main characteristics of the studies are summarized in Table 1. The selected publications represent a total of 3372 enrolled patients. Out of them, 1773 (52.58%) were part of randomized clinical trials. The sample sizes of the studies varied (50–802 patients). The mean age of the patients in 10 of the publications [7,43,44,45,46,47,48,49,50,51] was 71.85 ± 22.09 years (minimum age 12; maximum age 88). The major indication for pacemaker implantation was atrioventricular block [7,44,45,46,47,48,49,51]. The study period ranged from 4 weeks [7] to 372 months [48]. All of the selected studies used the same pacemaker model in both follow-up arms, with the exception of the studies by Folino et al. [45,46] and Lopez-Villegas et al. [50], who used two different pacemaker models in the CM group. None of the selected studies stated if monitoring systems were previously being used for all pacemakers followed up by the hospital. A cost-utility analysis was performed in five of the publications [7,48,49,50,51].

### 3.2. Health Variables Analysis

Table 2 contains the primary and secondary endpoints analyzed in each of the studies, as well as the most significant results. Only the studies of Folino et al. [45,46] included the number of pacemaker replacements (ranging from 7 to 123), and the reported device longevity ranged from 6.7 years to 8.3 years [7,44,45,46]. Only two studies [7,48] specified the mean hospital stay, which was 34% to 73.2% shorter in the TM group. Besides, two studies [7,48] administered the SF-36 questionnaire, and the other three studies [49,50,51] used the EuroQol–5D (EQ–5D) questionnaire to evaluate the health-related quality of life (HRQoL). The results indicated no significant differences between the two alternatives of follow-up.

Out of the eight studies that included information on adverse events per year [7,42,43,44,45,46,47,48], six of them reported a higher percentage of events in the TM group [7,42,43,44,45,46]; the study by Folino et al. [46] reported the highest percentage (52%). In contrast, only one study [48] reported a higher percentage of events in the CM group (35.40%) as compared to the TM group (21.70%). The percentage of patients in the active group, who had to visit the hospital so that their pacemaker could be reprogrammed [46], ranged from 0.6 to 1.9% per year. In seven of the 11 studies included in this systematic review (63.64%), the annual mortality rate [7,42,44,45,46,50,51] ranged from 0 to 11.7%.

### 3.3. Cost Analysis

The costs of implementing TM in patients with pacemakers were not included in any of the studies selected in this systematic review (Table 2). Three of the papers stated that the costs of the “home monitoring system” (remote option) were paid by the hospital [43,49,50]. In contrast, in the articles written by Folino et al. [45,46], the costs of implementing TM systems were borne by the pacemaker manufacturers.

In order to facilitate the economic comparison of the different studies selected in this systematic review, the cumulative annual inflation was estimated from the year following the publication of the article to December 2020. Then, direct conversion of each currency to euros (€) based on the price on 12 August 2021, was made. The total costs of all the studies included in this review are lower in the TM group compared to that of the CM group, except in the results presented by Lopez-Villegas [50] (Table 2). In the WEST-SCOTLAND [42] study, the replacement of the CM modality of follow-up by the TM modality resulted in a saving of €14,669 per year (associated with ambulance transport) for the Scottish National Health System. A study carried out with a pediatric population [43] indicates that there would have been a saving of €18,611 over the 3 years of study period if the 96 participants had been able to substitute visits to the emergency room in the hospital with the data transmission system. The economic saving in the TM modality is evident in almost 82% (*n* = 9) of the selected studies [7,43,44,45,46,47,48,49,51], with the costs associated with the TM group being 9% to 86.69% lower than that of the CM group. In five (45.45%) of the selected studies [7,46,47,49,51], the patients in the TM group reported a reduced number of hospital visits (8.34–55.55%) compared to the patients in the CM group. The informal costs associated with each modality of follow-up (costs of transport, productivity, accompanying person, etc.) were estimated in five of the studies included in this systematic review [45,48,49,50,51], which indicates that in the remote modality of follow-up, cost savings of up to 56.70% per patient/year can be achieved [49]. Table 3 shows the costs of the follow-up alternatives included in this systematic review.

### 3.4. Methodological Quality Assessment

The variables evaluated were scored based on the presence or absence (yes/no answers) of the criterion analyzed (Table 4). If, on the final review of the article, a parameter was not found, a response of “no” was recorded in the table, i.e., the study did not include that parameter.

The study by Bautista-Mesa et al. [51] obtained the highest overall score for methodological quality, with 24 out of a possible 25 points, whereas the lowest score of 7 was obtained in the study by Shaw [42]. The publications evaluated had a mean score of 15.55 ± 5.07 points (minimum 7; maximum 24). The main findings were as follows:

(a) Five manuscripts [45,47,49,50,51] included results with both social and financial perspectives (NHS);
(b) Five studies [7,48,49,50,51] have used social assessment scales for evaluating HRQoL, which were validated on a representative sample of the population;
(c) Except for one study [51], none of the studies applied modeling techniques or discounts for costs and benefits or conducted a sensitivity analysis;
(d) The results obtained from eight of the selected studies [7,44,45,47,48,49,50,51] could draw conclusions about the transferability or extrapolation of results to other contexts;
(e) The results of all the included studies are presented with an incremental analysis; however, the results of three studies are disaggregated (costs and results of the alternatives) [49,50,51];
(f) Five of the studies [7,42,43,44,49,50,51] have clearly indicated the financial source of the study.

### 3.5. Intercoder Reliability

The results for each variable are shown in Table 5. Mean and standard deviation values were not calculated because the variables included in this study were categorical. The study by Vincent et al. [43] obtained the lowest percentage agreement of 84%, with other parameters being Scott’s pi −0.087, Cohen’s kappa −0.087, and Krippendorff’s alpha −0.065; the highest percentage agreement of 100% was obtained in four studies [44,45,46,51].

## 4. Discussion

The results of this review indicate no significant differences in HRQoL and the number of cardiovascular events between TM and CM modalities of the follow-up. The results show that TM was significantly effective in detecting the presence or absence of pacemaker problems, leading to a reduction in the number of unscheduled hospital visits. In addition, follow-up costs in the remote modality are significantly lower than that of the CM modality. The economic impact of each of the monitoring alternatives is significantly influenced by the costs associated with staff salaries, transport, informal care, and absences from work.

### 4.1. Effectiveness and Clinical Safety of TM Systems

Four of the 11 studies analyzed in this systematic review (36.37%) included the data regarding the number of hospital visits made by patients in both follow-up alternatives [7,47,49,51]. The main finding was that in the TM group, there was a significant reduction of 8.34–55.55% in the number of hospital visits. These results were similar (in the upper range) to those previously published in the COMPAS study [19], which reported that patients of the TM group made 55% fewer hospital visits compared to patients included in the CM group. In contrast, three of the articles (27.27%) [43,44,45] reported results pertaining to the TM group only.

The development and expansion of remote pacemaker monitoring systems have proven that this is a safe and reliable technology [7,42,43,44,45,46,47,48,49,50,51]. The steady increase in the transmission of information from the patient’s home monitor to the service provider’s platform has enabled quick and efficient treatment of cardiac patients on an ongoing basis. It is also noted that in the medium to long term, there is a significant reduction in the number of unscheduled visits and/or hospitalizations. In four of the publications included in this review [7,46,47,48], there were no significant differences between the two follow-up modalities in relation to the number of adverse events detected, which is in accordance with two previously published studies [19,22]. In a previous study on pacemakers [16], it is reported that cardiovascular events were detected around two months earlier in the TM group (5.7 vs. 7.7). In two subsequent studies carried out on patients with implantable cardioverter-defibrillator and cardiac resynchronization therapy, the response time to these episodes was 22–36 days in the case of the CM group; however, in the TM group, it was reduced to 2–4.6 days [22,53].

Five of the selected studies analyzed HRQoL of the included patients (45.46%) [7,48,49,50,51]. The SF-36 questionnaire was used in two studies [7,48] and the EQ-5D was used by Lopez-Villegas et al. [49,50] and Bautista-Mesa et al. [51]. The results indicate no significant differences between the two follow-up modalities in all patients. These results coincide with those found in the COMPAS [19] and ECOST [22] trials, which used the SF-36 questionnaire, and with the PONIENTE [20] study, which used the EQ-5D questionnaire.

The analysis of the methodological quality of the manuscripts included in this study exhibited significant variability among them, with higher scores obtained by the most recent studies [49,50,51]. The results presented in this systematic review, which coincide with a previous study [54] published in 2016, indicate how difficult it is to assess the methodological quality of studies published in the last two decades based on the current criteria [42,43]. However, different inputs are included in all the selected studies, such as the establishment of an objective and research question, comparison of both follow-up modalities, adjustment of the costs collected to the perspective of the selected analysis, and adaptation of the time horizon to the study objectives. Additionally, they coincide, except for the study by Bautista-Mesa et al. [51], in not implementing modeling techniques, discounting costs, performing sensitivity analysis, justifying key parameters and statistical distribution of the variables, performing equity analysis, and including cost-effectiveness and cost-benefit ratios.

### 4.2. Cost Analysis

The results presented in this systematic review confirm that TM of pacemakers can significantly decrease the length of hospital stays [7,48] reaching in some cases a reduction of up to 80.49%. One of the most significant findings is the substantial reduction of 9% to 86.69% [7,42,43,44,45,46,47,48,49,50,51] in the costs of TM with respect to that of CM.

In addition, the results of most of the studies included in this review indicate that TM systems significantly reduce direct costs, such as for staff and health administration, as well as indirect costs related to monitoring, such as transport costs, maintenance of consultations, and waiting rooms, etc. The results found in this systematic review are similar to those obtained in previous studies that were carried out on different types of cardiovascular electronic devices [2,15,26,27,55], and on remote follow-up performed in other pathologies, such as rheumatoid arthritis [56], mental health [57], teleglaucoma [58], teledermatology, and tele radiology [59,60,61]. In a study published in 2009 by Raatikainen, it was reported that a lower number of hospital visits resulted in up to 41% reduction in costs per patient [27]. A study published by Elsner [62] reported a 61% increase in savings due to a reduction of 63% in the number of visits and the transport costs associated with them. Finally, and coinciding with the results obtained in this review, Crossley published a study reporting that reducing the number of days spent in hospitals can achieve savings of almost $1700 per patient per year [16].

### 4.3. Study Limitations

Although the results presented in this systematic review are highly relevant in relation to the effectiveness of TM in patients with pacemakers, the analysis carried out presents several limitations that should be taken into account.

First, the number of included studies (*n* = 11) and enrolled participants (*n* = 3427) were less, mainly due to the limited use of TM technology compared to CM. The second limitation is the variability in the methodological quality of the selected studies; except for one study [51], none of them used modeling techniques and discounts in costs and results. Apart from this, the key parameters of the study and the statistical distribution of the variables examined in the sensitivity analysis were not explained properly. The third limitation is the small number of studies [48,51] analyzing the medium-and long-term effectiveness of remote monitoring, as TM is a relatively new technology. The fourth limitation is the large time span of 39 years between the first and the last published study [42,51], during which, exponential changes in these technologies have occurred. Furthermore, in this study, the differences and similarities between both monitoring modalities have been verified in different spatiotemporal contexts. Finally, cost-effectiveness studies were less generalizable compared to effectiveness studies since they depend on both the duration and the context in which the studies are carried out; yet their importance is enormous since they facilitate decision-making by the different professionals involved [54]. This systematic review presents the significant results of studies carried out in the last 40 years (1981–2020), mainly focusing on the health outcomes and costs associated with TM of patients with pacemakers. Therefore, the findings of this systematic review have led to an update in scientific knowledge in this area, and the results can be further utilized to facilitate decision-making and the implementation of new health policies. The authors of this study advise future researchers to focus on economic evaluations, comparing both follow-up modalities, including the cost-effectiveness ratios and the informal costs associated with the follow-up. In addition, the time horizon should be medium and long term.

The results presented in this study can be used by both healthcare managers and cardiology unit professionals to promote the sustainability of current healthcare systems.

## 5. Conclusions

Most of the studies included in this systematic review confirm that in the TM of patients with pacemakers, there is a reduction in cardiovascular events and hospital visits without affecting the HRQoL of patients. In addition, both formal and informal costs are significantly reduced in the medium and long term.

## Figures and Tables

**Table 1:** Study characteristics.

Reference, Country	Follow-Up, Months	Design	Sample, *n*; (Age, y)	Men, %	TM Used	Inclusion Criteria	Exclusion Criteria	Type of Analysis	Perspective	Costs Evaluated
Shaw et al. [42], 1981 United Kingdom	12	Multicenter clinical trial	783; (N/A)	N/A	TTM Cardiotrak W System	Have a PM implanted	N/A	CEA	NHS	Direct and indirect
Vincent et al. [43], 1997 USA	36	Single-center observational	96; (12)	N/A	TTM Medtronic Teletrace model 9431	(1) Have a PM implanted, (2) congenital, (3) idiopathic symptomatic sinus dysfunction or AVB node dysfunction	N/A	CEA	NHS	Direct
Halimi et al. [7], 2008 France-Belgium	1	Randomized, open-label, parallel-and non-inferiority multicenter clinical trial	379; (75)	61	Biotronik HM^®^	(1) >18 y, PM implant, (2) comply with protocol/sign IC; (3) clinically stable; (4) discharged from hospital within 24 h after implantation	(1) Spontaneous ventricular rate < 30 b.p.m., (2) overt heart failure, (3) history of cardiac surgery or myocardial infarction within 1 month, (4) were systemically anticoagulated, (5) unable to understand TM, no access to GSM	CUA	NHS	Direct and indirect
Pang et al. [44], 2010 Canada	10	Single-center observational	303; (82)	49	TTM Instromedix LifeSigns W	N/A	N/A	CBA	NHS	Direct and indirect
Folino et al. [45], 2012 Italy	80	Single-center observational	802; (88)	39	Biotronik HM^®^	Patients with in-home Biotronik PM	N/A	CMA	Hospital, patients and NHS	Direct and indirect
Folino et al. [46], 2013 Italy	27	Single-center observational	398; (88)	63	Medtronic CareLink^®^ Network (Medtronic)	(1) Severe limitation in walking; (2) transported in ambulance; (3) implantation of PM compatible with Carelink^®^ TM system; (4) availability of a telephone landline; (5) life-expectancy > 6 months	N/A	CEA	NHS	Direct
Perl et al. [47], 2013 Austria	27	Single-center clinical trial	115; (74)	60	Biotronik^®^ System	(1) Double chamber PM implantation; (2) Geographical and medically stable; (3) GSM coverage	N/A	CEA	NHS and Social	Direct and indirect
Parahuleva et al. [48], 2017 Germany	372	Retrospective, single-center, parallel, noninferiority case series study	364; (65.5)	76	Biotronik HM system^®^	(1) age > 18 years, (2) indication for first implant of CIEDs, (3) stable medical status, and (4) the ability to discharge the patient from the hospital within 24 h after first device implant.	(1) had a spontaneous ventricular rate < 30 bpm, (2) were in overt heart failure, (3) had a history of cardiac surgery or myocardial infarction within 1 month, (4) were systemically anticoagulated, (5) were unable to understand the TM system, (6) were pregnant or breastfeeding, or (7) they were unwilling to provide written informed consent to participate.	CUA	NHS	Direct
Lopez-Villegas et al. [49], 2019 Spain	12	Controlled, non-randomized, non-masked single-center clinical trial	82; (78)	78	Medtronic CareLink^®^	(1) >18 y, PM implant, (2) comply with protocol/sign IC; (3) capable of understanding and correctly performing the home auto-monitoring or had a caregiver who could carry out this function.	(1) Patients enrolled in another study; (2) other cardiac device; (3) refuse to participate.	CUA	NHS and Social	Direct and indirect
Lopez-Villegas et al. [50], 2020 Spain	12	Controlled, randomized, non-masked single-center clinical trial	50; (75)	52	Biotronik Estella SR-T/DR-T^®^//Biotronik Evia SR-T/DR-T^®^	(1) >18 y, (2) PM implant, (3) comply with protocol/sign IC; (4) capable of understanding and correctly performing the home auto-monitoring or had a caregiver who could carry out this function.	(1) Patients enrolled in another study; (2) other cardiac device; (3) refuse to participate).	CUA	NHS and Social	Direct and indirect
Bautista-Mesa et al. [51], 2020 Spain	360	Controlled, non-randomized, non-masked single-center clinical trial	55; (81)	69	Medtronic CareLink^®^	(1) > 18 y, (2) PM implant, (3) comply with protocol/sign IC; (4) capable of understanding and correctly performing the home auto-monitoring or had a caregiver who could carry out this function [20,21].	(1) Patients enrolled in another study; (2) other cardiac device; (3) refuse to participate [20,21].	CUA	NHS and Social	Direct and indirect

AVB, atrioventricular block; bpm, beats per minute; CBA, cost-benefit analysis; CEA, cost-effectiveness analysis; CMA, cost-minimization analysis; CUA, cost-utility analysis; GSM, global system for mobile communications; HM^®^, Home Monitoring^®^; IC, informed consent; N/A, not available; NHS, national health system; PM, pacemaker; TM, telemonitoring; TTM, transtelephonic monitoring.

**Table 2:** Analysis of main outcomes, inputs, and conclusions.

Reference, Country	Primary Outcomes	Secondary Outcomes	No. of Hospitalizations		Follow-Ups/Patient/Year		Adverse Events/Year		Visits to Emergency Service		Annual Mortality		Analysis of Cost/Year *** € Value = 2021		Conclusions
			CM	TM	CM	TM	CM	TM	CM	TM	CM	TM	CM	TM	
Shaw et al. [42], 1981 United Kingdom	Cost savings for traveling patients	Clinic visits, effective changes of generator, generator failures, reoperations, emergency admissions, deaths, health care costs	N/A	1	N/A	N/A	N/A	1	N/A	1	3.7% mortality from both groups		Annual saving on transport: €14,669		TM of patients with pacemakers is carefully monitored to ensure that they receive adequate attention without any inconvenience.
Vincent et al. [43], 1997 USA	Diagnostic capabilities	Cost-effectiveness of TM	N/A	N/A	N/A	4.76	N/A	1%	N/A	N/A	N/A	N/A	TM conferred an annual saving of: €20,450/€18,611		TM was significantly effective in detecting the presence or absence of pacemaker problems. Financial charges involved were significantly less compared to outpatient visits.
Halimi et al. [7], 2008 France-Belgium	Rate of MAEs	Detection of pacing system dysfunction, duration of hospitalizations, cost saving, and quality of life	4.8	3.2	7.1	5.92	19.0%	20.1%	N/A	N/A	1	0	€8000	€7688	Early discharge of patients after pacemaker implantation followed by TM was safe and facilitated the monitoring of patients in the month following the procedure.
Pang et al. [44], 2010 Canada	TM effectiveness and feasibility	Extrapolate the costs of CM to TM	N/A	N/A	N/A	4.7	4.1%	5.3%	N/A	N/A	12 deaths from both groups		€84,210	€11,209	Apart from reducing the costs involved in conventional follow-up of patients, TM is considered safe and permits follow-up of patients who have difficulty visiting the clinic.
Folino et al. [45], 2012 Italy	Efficacy and reliability	Healthcare and informal costs	N/A	N/A	N/A	0.45	N/A	0.30	N/A	N/A	8.7% from both groups		€73.84	€61.26	TM modality is as safe and reliable as CM modality. Besides, costs were 20.5% lower than the former.
Folino et al. [46], 2013 Italy	Longevity, ECG and technical data from PM	Costs of a system for TM of PM	N/A	N/A	1.3	2.6	N/A	52%	N/A	N/A	8.3%	11.7%	€79.64	€40.21	TM of pacemaker is a reliable, effective, and cost-saving procedure in elderly, debilitated patients. Moreover, remote
															controls provided an accurate and early diagnosis of arrhythmia occurrence.
Perl et al. [47], 2013 Austria	Costs and number of hospital visits	Safety of TM	15	11	0.53	0.29	No significant differences		N/A	N/A	N/A	N/A	TM was 58.7% cheaper than CM		TM was safe, reduced overall hospital visits, and detected events that mandated unscheduled visits.
Parahuleva et al. [48], 2017, Germany	HRQoL	Healthcare and informal costs	N/A	N/A	N/A	N/A	35.40%	21.70%	N/A	N/A	N/A	N/A	Costs are 22–25% lower for patients assigned to the TM Group		TM was safe and not inferior to the classic medical procedure. Besides, it involves lower costs.
Lopez-Villegas et al. [49], 2019, Spain	HRQoL	Healthcare and informal costs	0	1	3.92	2.87	N/A	N/A	N/A	N/A	N/A	N/A	€187.02	€79.93	TM appears to be a significant cost-effective alternative to CM for both healthcare workers and patients.
Lopez-Villegas et al. [50], 2020, Spain	HRQoL	Healthcare and informal costs	0	3	1.56	1.56	ND	ND	D	ND	2	2	€442.43	€2360	Cost-utility analysis of TM vs. CM indicates inconclusive results because of broad confidence intervals, with ICER and INB figures ranging from potential savings to high costs for
															an additional QALY. The majority of ICERs are above the usual NHS thresholds for coverage decisions.
Bautista-Mesa et al. [51], 2020, Spain	HRQoL	Healthcare and informal costs	ND	ND	1.49	0.88	ND	ND	ND	ND	2.8	1.6	€366.60	€282.20	TM of older patients with pacemakers appears to be a costly alternative to CM after five years of follow-up.

ECG, electrocardiogram; MAE, major adverse event; N/A, not available; PM, pacemaker; TM, telemonitoring. *** inflation calculator: https://fxtop.com/ (accessed on 12 August 2021).

**Table 3:** Costs evaluated in both modalities of follow-up.

Reference, Country	Telemonitoring	Conventional Monitoring
Shaw et al. [42], United Kingdom	✓ Staff ✓ Telephone ✓ Transport	✓ Staff ✓ Telephone ✓ Transport
Vincent et al. [43], United States	✓ Monthly cost of routine and emergency visits (including PM analysis) ✓ Emergency department costs excluding PM analysis	✓ Monthly cost of routine and emergency visits (including PM analysis) ✓ Emergency department costs excluding PM analysis
Halimi et al. [7], France/Belgium	✓ Staff ✓ Laboratory ✓ Indirect costs (physicians and paramedics) ✓ Transport	✓ Staff ✓ Laboratory ✓ Indirect costs (physicians and paramedics) ✓ Transport
Pang et al. [44], Canada	✓ Staff (nurses) ✓ Hospital visits ✓ Equipment rental ✓ Telephone calls	✓ Staff (physician + nurse) ✓ Hospital services ✓ Allowances ✓ Transport costs
Folino et al. [45], Italy	✓ Health care costs (physician, nurse, and transport) ✓ Informal costs (transport and productivity) ✓ NHS (PM check costs)	✓ Health care costs (physician, nurse, and transport) ✓ Informal costs (transport and productivity) ✓ NHS (PM check costs)
Folino et al. [46], Italy	✓ NHS (visit costs) ✓ Staff (physician + nurse) ✓ Transport	✓ NHS (visit costs) ✓ Staff (physician + nurse) ✓ Transport
Perl et al. [47], Austria	✓ Staff ✓ Transport	✓ Staff ✓ Transport
Parahuleva et al. [48], Germany	✓ Health care costs (consultation fee for cardiologist) ✓ Biotronik service center.	✓ Health care costs (consultation fee for cardiologist) ✓ Biotronik service center.
Lopez-Villegas et al. [49], Spain	✓ Healthcare costs (hospital staff costs, consultation room costs, ambulance costs, hospitalization costs) ✓ Social costs (patients‘ perspective: accompanying person, travel per patient-year)	✓ Healthcare costs (hospital staff costs, consultation room costs, ambulance costs, hospitalization costs) ✓ Social costs (patients‘ perspective: accompanying person, travel per patient-year)
Lopez-Villegas et al. [50], Spain	✓ Healthcare costs (hospital staff costs, consultation room costs, ambulance costs, hospitalization costs) ✓ Social costs (patients‘ perspective: accompanying person, travel per patient-year)	✓ Healthcare costs (hospital staff costs, consultation room costs, ambulance costs, hospitalization costs) ✓ Social costs (patients ‘perspective accompanying person, travel per patient-year)
Baustista-Mesa et al. [51], Spain	✓ NHS perspective: staff costs, consultation room costs, ambulance costs ✓ patients’ perspective: informal transport, lost income	✓ NHS perspective: staff costs, consultation room costs, ambulance costs ✓ Patients’ perspective: informal transport, lost income

NHS, National Health System; PM, pacemaker.

**Table 4:** Checklist for analyzing methodological quality of the studies.

	Shaw et al., 1981 [42]	Vincent, et al., 1997 [43]	Halimi et al., 2008 [7]	Pang et al., 2010 [44]	Folino et al., 2012 [45]	Folino et al., 2013 [46]	Perl et al., 2013 [47]	Parahuleva et al., 2017 [48]	Lopez-Villegas et al., 2019 [49]	Lopez-Villegas et al., 2020 [50]	Bautista-Mesa et al., 2020 [51]
1. Did the study clearly establish the aims and the research question?	Yes	Yes	Yes	Yes	Yes	Yes	Yes	Yes	Yes	Yes	Yes
2. Was the economic evaluation done in a general manner and later in population subgroups (age, sex, severity, and levels of risk). Does the data indicate relevant differences in the cost or effectiveness between them?	No	No	Yes	Yes	Yes	Yes	Yes	Yes	Yes	Yes	Yes
3. Did the economic evaluation include the social perspective as well as the financial perspective (NHS)?	No	No	Yes	No	Yes	Yes	Yes	No	Yes	Yes	Yes
4. Are both perspectives reported separately and clearly differentiated?	No	No	No	No	Yes	No	Yes	No	Yes	Yes	Yes
5. Was the technology compared with at least one routine clinical practice?	Yes	Yes	Yes	Yes	Yes	Yes	Yes	Yes	Yes	Yes	Yes
6. Is the choice of comparison option clearly explained?	Yes	No	Yes	Yes	Yes	Yes	Yes	Yes	Yes	Yes	Yes
7. Is the type of analysis chosen sufficiently explained in relation to the original question?	No	No	Yes	No	Yes	Yes	Yes	Yes	Yes	Yes	Yes
8. Is the source used to obtain efficacy or effectiveness data explained in detail?	No	No	Yes	Yes	Yes	Yes	Yes	Yes	Yes	Yes	Yes
9. Are the design and methods explained in detail?	No	No	Yes	No	Yes	Yes	Yes	Yes	Yes	Yes	Yes
10. Were the selected outcome measures clinically relevant (final efficacy/effectiveness measurement)?	No	Yes	Yes	Yes	Yes	Yes	Yes	Yes	Yes	Yes	Yes
11. Have the social scales for assessment of health-related quality of life (HRQoL) been validated based on a sample that is representative of the population?	No	No	Yes	No	No	No	No	Yes	Yes	Yes	Yes
12. Were the reported costs adjusted to the selected analysis perspective?	Yes	Yes	Yes	Yes	Yes	Yes	Yes	Yes	Yes	Yes	Yes
13. Were the physical units of the costs and the cost data separated and explained in adequate detail?	Yes	Yes	No	No	Yes	Yes	Yes	No	Yes	Yes	Yes
14. Was the time horizon the most appropriate to pick up all the differential effects of the evaluated technology on health and the resources used?	Yes	Yes	Yes	Yes	Yes	Yes	Yes	Yes	Yes	Yes	Yes
15. If modelling techniques were used, are the choice of model, the parameters, and the key assumptions explained and transparent?	No	No	No	No	No	No	No	No	No	No	Yes
16. Were costs and future results discounted using the same rates?	No	No	No	No	No	No	No	No	No	No	Yes
17. Was a sensitivity analysis performed?	No	No	No	Yes	No	No	No	No	No	No	Yes
18. Are the key parameters of the study and the statistical distribution of the variables analyzed in the sensitivity analysis explained?	No	No	No	No	No	No	No	No	No	No	Yes
19. If arguments of social justice were included in the evaluation (fairness analysis), is this analysis presented separately from the main evaluation, and are the arguments used transparent?	No	No	No	No	No	No	No	No	No	No	No
20. Does the report allow conclusions to be drawn on the transferability or extrapolation of results to other contexts?	No	No	Yes	Yes	Yes	No	Yes	Yes	Yes	Yes	Yes
21. Are the results presented with an incremental analysis and also broken down (costs and results of the alternatives)?	No	No	No	No	No	No	No	No	Yes	Yes	Yes
22. Are the limitations or weak points of the analysis presented in a critical and transparent manner?	No	No	Yes	Yes	No	Yes	Yes	Yes	Yes	Yes	Yes
23. Do the conclusions of the study answer the original question and were they clearly derived from the results obtained?	Yes	Yes	Yes	Yes	Yes	Yes	Yes	Yes	Yes	Yes	Yes
24. Is it clearly stated who led, supported, or financed the study?	Yes	No	Yes	No	No	No	No	No	Yes	Yes	Yes
25. Are possible conflicts of interest stated?	No	No	Yes	No	Yes	Yes	No	Yes	Yes	Yes	Yes
TOTAL	8	7	17	12	16	15	16	16	20	20	24

NHS, National Health System; No, absence of criterion; Yes, presence of criterion.

**Table 5:** Intercoder reliability and percentages.

Variable	Percent Agreement (%)	Scott’s Pi	Cohen’s Kappa	Krippendorff’s Alpha (Nominal)	Agreements (*n*)	Disagreements (*n*)	Cases (*n*)	Decisions (*n*)
Shaw et al. [42], (cols 1 and 2)	92	−0.042	0	−0.021	23	2	25	50
Vincent et al. [43], (cols 1 and 2)	84	−0.087	−0.087	−0.065	21	4	25	50
Halimi et al. [7], (cols 1 and 2)	96	−0.02	0	0	24	1	25	50
Pang et al. [44], (cols 1 and 2)	100	1	1	1	25	0	25	50
Folino et al. [45], (cols 1 and 2)	100	1	1	1	25	0	25	50
Folino et al. [46], (cols 1 and 2)	100	1	1	1	25	0	25	50
Perl et al. [47], (cols 1 and 2)	96	−0.02	0	0	24	1	25	50
Parahuleva et al. [48], (cols 1 and 2)	96	−0.02	0	0	24	1	25	50
Bautista-Mesa et al. [49], (cols 1 and 2)	96	−0.02	0	0	24	1	25	50
Lopez-Villegas et al. [50], (cols 1 and 2)	96	−0.02	0	0	24	1	25	50
Bautista-Mesa et al. [51], (cols 1 and 2)	100	1	1	1	25	0	25	50

## Data Availability

The datasets used and/or analyzed during the current study are available from the corresponding author on reasonable request.

## Supplementary Materials

The following are available online at https://www.mdpi.com/article/10.3390/ijerph182212120/s1, File S1: References not included in the systematic review.
