# Supplementary material for: Knowledge Update on the Economic Evaluation of Pacemaker Telemonitoring Systems
Source: Int J Environ Res Public Health. 2021 Nov 18;18(22):12120. doi: 10.3390/ijerph182212120 (PMC8624333; doi:10.3390/ijerph182212120)
Supplement: Supplementary file 1 [file ijerph-18-12120-s001.zip › ijerph-1444832-supplementary.pdf]

REFERENCES NOT INCLUDED IN THE SYSTEMATIC REVIEW

File S1. References not included in the systematic review.

1. Eduardo G. Bertoldi
2. Search for articles by this author
3. Affiliations
4. Postgraduate Program in Cardiology and Cardiovascular Sciences, Federal University of Rio Grande do Sul, Porto Alegre, Brazil

1. Al-Khatib SM, Piccini JP, Knight D, Stewart M, Clapp-Channing N, Sanders GD. Remote monitoring of implantable cardioverter defibrillators versus quarterly device interrogations in clinic: results from a ramdomized pilot clinical trial. J Cardiovasc Electrophysiol. 2010;21(5):545-50.
2. Al-Khatib SM, Mi X, Wilkoff BL, Qualls LG, Frazier-Mills C, Setoguchi S, et al. Follow-up of Patients With New Cardiovascular Implantable Electronic Devices. Are Experts' Recommendations Implemented in Routine Clinical Practice?. Circ Arrhythm Electrophysiol. 2013;6(1):108-16.
3. Al-Razzo O, Gonzáles-Villegas E, Silvestre-García J, Cabestrero-Alonso D, Represa-Pastor T, Alejandre-Leyva M, et al. Seguimiento domiciliario como sustitutivo de seguimiento presencial en pacientes portadores de marcapasos. Cuadernos de Estimulación Cardíaca. 2011;10(4):57-62.
4. Biffi M, Bertini M, Saporito D, Ziacchi M, Martignani C, Diemberger I et al. Actual pacemaker longevity: the benefit of stimulation by automatic capture verification. Pacing Clin Electrophysiol. 2010;33(7):873-81.
5. Boriani G, Auricchio A, Klersy C, Kirchhof P, Brugada J, Morgan J et al. Healthcare personnel resource burden related to in-clinic follow-up of cardiovascular implantable electronic devices: a European Heart Rhythm Association and Eucomed joint survey. Europace. 2011;13(8):1166-73.
6. Boriani G, Diemberger I, Biffi M, Martignani C. Cost-effectiveness of cardiac resynchronisation therapy. Heart 2012;98(24):1828-36.
7. Brugada P. What evidence do we have to replace in-hospital implantable cardioverter defibrillator follow-up? Clin Res Cardiol. 2006;95 Suppl 3:III3-9.
8. Burri H, Senouf D. Remote monitoring and follow-up of pacemakers and implantable cardioverter defibrillators. Europace. 2009;11(6):701–9.
9. Burri H, Heidbüchel H, Jung W, Brugada P. Remote monitoring: a cost or an investment? Europace. 2011;13 Suppl 2:ii44-8.
10. Calcagnini G, Censi F, Floris M, Pignalberi C, Ricci R, Biancalana G et al. Evaluation of electromagnetic interference of GSM mobile phones with pacemakers featuring remote monitoring functions. Pacing Clin Electrophysiol. 2006;29(4):380-5.
11. Calisto J, Faria H, Pego G, Angelo FA, Rufino E, Raposo A et al. A follow-up by telephone of patients wearing a pacemaker--the experience of the Cardiac Pacing Center of the Hospitais de Universidade de Coimbra. Rev Port Cardiol. 1993;12(6):551-5.
12. Capucci A, Santini M, Padeletti L, Gulizia M, Botto G, Boriani G et al. On behalf of the Italian AT500 Registry. Monitored atrial fibrillation duration predicts arterial embolic events in patients suffering from bradicardia and atrial fibrillation implanted with antitachycardia pacemakers. J Am Coll Cardiol 2005;46(10):1913-20.
13. Caro J, Ward A, Moller J. Modelling the health benefits and economic implications of implanting dual-chamber vs. single-chamber ventricular pacemakers in the UK. Europace. 2006;8(6):449-55.
14. Chronaki CE, Vardas P. Remote monitoring costs, benefits, and reimbursement: a European perspective. Europace. 2013;15 Suppl 1:i59-i64.
15. Cronin E, Varma N. Remote monitoring of cardiovascular implanted electronic devices: a paradigm shift for the 21st century. Expert Rev Med Devices. 2012;9(4):367-76.
16. Cronin EM, Ching EA, Varma N, Martin DO, Wilkoff BL, Lindsay BD. Remote monitoring of cardiovascular devices: a time and activity analysis. Heart Rhythm 2012;9:1947–1951.

REFERENCES NOT INCLUDED IN THE SYSTEMATIC REVIEW

17. Crossley GH, Chen J, Choucair W, Cohen TJ, Gohn DC, Johnson WB et al. Clinical benefits of remote versus transtelephonic monitoring of implanted pacemakers. J Am Coll Cardiol, 2009;54(22):2012-9.
18. Crossley GH, Boyle A, Vitense H, Chang Y, Mead RH; CONNECT Investigators. The CONNECT (Clinical Evaluation of Remote Notification to Reduce Time to Clinical Decision) trial: the value of wireless remote monitoring with automatic clinician alerts. J Am Coll Cardiol. 2011;57(10):1181-9.
19. De Cock C, Elders J, van Hemel M, van den Broek K, van Erven L, de Mol B et al. Remote monitoring and follow-up of cardiovascular implantable electronic devices in the Netherlands. An expert consensus report of the Netherlands Society of Cardiology. Neth Heart J. 2012;20(2):53-65.
20. Deniz HB, Caro JJ, Ward A, Moller J, Malik F. Economic and health consequences of managing bradicardia with dual-chamber compared to single-chamber ventricular pacemakers in Italy. J Cardiovasc Med. 2008;9(1):43-50.
21. Drak-Hernández Y, Toquero-Ramos J, Fernández JM, Pérez-Pereira E, Castro-Urda V, Fernández-Lozano I. Effectiveness and safety of remote monitoring of patients with an implantable loop recorder. Rev Esp Cardiol (Engl Ed). 2013;66(12):943-8.
22. Dreifus LS, Zinberg A, Hurzeler P, Puziak AD, Pennock R, Feldman M et al. Transtelephonic monitoring of 25,919 implanted pacemakers. Pacing Clin Electrophysiol. 1986;9(3):371-8.
23. Dubner S, Auricchio A, Steinberg J, Vardas P, Stone P, Brugada J et al. ISHNE/EHRA expert consensus on remote monitoring of cardiovascular implantable electronic devices (CIEDs). Europace. 2012;14(2):278-93.
24. Elsner CH, Sommer P, Piorkowski C, Taborsky M, Neuser H, Bytesnik J et al. A prospective multicenter comparison trial of home monitoring against regular follow-up in MADIT II patients: additional visits and cost impact. Comput Cardiol. 2006;33:241-4.
25. Eze ND, Mateus C, Cravo Oliveira Hashiguchi T. Telemedicine in the OECD: An umbrella review of clinical and cost-effectiveness, patient experience and implementation. PLoS One. 2020;15(8):e0237585.
26. Fanourgiakis J, Simantirakis E, Maniadakis N, Kourlaba G, Kanoupakis E, Chrysostomakis S et al. Cost-of-illness study of patients subjected to cardiac rhythm management devices implantation: results from a single tertiary centre. Europace. 2013;15(3):366-75.
27. Fauchier L, Sadoul N, Kouakam C, Briand F, Chauvin M, Babuty D et al. Potential cost savings by telemedicine-assited long-term care of implantable cardioverter defibrillator recipients. Pacing Clin Electrophysiol. 2005;28 Suppl 1:S255-9.
28. Feldman AM, de Lissovoy G, Bristow MR, Saxon LA, De Marco T, Kass DA et al. Cost effectiveness of cardiac resynchronization therapy in the Comparison of Medical Therapy, Pacing, and Defibrillation in Heart Failure (COMPANION) trial. See comment in PubMed Commons belowJ Am Coll Cardiol. 2005;46(12):2311-21.
29. Folino AF, Chiusso F, Zanotto G, Vaccari D, Gasparini G, Megna A et al. Management of alert messages in the remote monitoring of implantable cardioverter defibrillators and pacemakers: an Italian single-region study. Europace. 2011;13(9):1281-91.
30. Furman S, Escher DJ. Transtelephone pacemaker monitoring: five years later. Ann Thorac Surg. 1975;20(3):326-38.
31. García Urra F, Porres Aracama JM, Fontán Martín-Chico B. Dispositivos eléctricos y monitorización remota. Rev Urug Cardiol. 2013; 28(1):63-70.
32. Gessman LJ, Vielbig RE, Waspe LE, Moss L, Damm D, Sundeen F. Accuracy and clinical utility of transtelephonic pacemaker follow-up. Pacing Clin Electrophysiol. 1995;18(5 Pt 1):1032-6.
33. Gillis AM. Remote Monitoring of Implantable Defibrillators: Reducing Hospitalizations and Saving Lives? Circ Arrhythm Electrophysiol. 2015;8(5):1010-1.
34. Gramegna L, Tomasi C, Gasparini G, Scaboro G, Zanon F, Boaretto G et al. In-hospital follow-up of implantable cardioverter defibrillator and pacemaker carriers: patients' inconvenience and points of view. A four-hospital Italian survey. Europace. 2012;14(3):345-50.

REFERENCES NOT INCLUDED IN THE SYSTEMATIC REVIEW

35. Guédon-Moreau L, Lacroix D, Sadoul N, Clémenty J, Kouakam C, Hermida JS et al. A randomized study of remote follow-up of implantable cardioverter defibrillators: safety and efficacy report of the ECOST trial. Eur Heart J. 2013;34(8):605-14.
36. Halimi F, Cantù F; European Heart Rhythm Association (EHRA) Scientific Initiatives Committee (SIC). Remote monitoring for active cardiovascular implantable electronic devices; a European survey. Europace 2010;12(12):1778-80.
37. Heerey A, Lauer M, Alsolaiman F, Czerr J, James K. Cost Effectiveness of Biventricular Pacemakers in Heart Failure Patients. Am J Cardiovasc Drugs. 2006;6(2):129-37.
38. Heidbuchel H, Lioen P, Foulon S, Huybrechts W, Ector J, Willems R et al. Potential role of remote monitoring for scheduled and unscheduled evaluations of patients with an implantable defibrillator. Europace. 2008(3);10:351-7.
39. Hummel JP, Leipold RJ, Amorosi SL, Bao H, Deger KA, Jones PW et al. Outcomes and costs of remote patient monitoring among patients with implanted cardiac defibrillators: An economic model based on the PREDICT RM database. J Cardiovasc Electrophysiol. 2019;30(7):1066-1077.
40. Irwin ME, Gulamhusein SS, Senaratne MP, St Clair WR. Outcomes of an Ambulatory Cardiac Pacing Program: Indications, Risks, Benefits, and Outcomes. Pacing Clin Electrophysiol. 1994;17(11 Pt 2):2027-31.
41. Joseph GK, Wilkoff BL, Dresing T, Burkhardt J, Khaykin Y. Remote interrogation and monitoring of implantable cardioverter defibrillators. J Interv Card Electrophysiol. 2004;11(2):161-6.
42. Kacet S, Guédon-Moreau L, Hermida JS et al, ECOST: safety of implantable cardioverter defibrillator follow-up using remote Monitoring a randomized controlled Trial (abstract). European society of Cardiology congress 2011.
43. Kim J, Beldner SJ, Jadonath R, Altman EJ. A Safe and Cost-Effective Approach to Treating Lyme Cardiac Disease in an Era of Health Care Reform. Pacing Clin Electrophysiol. 2011;34(6):666-9.
44. Kollmann A, Hayn D, García J, Trigo JD, Kastner P, Rotman B, Tscheliessnigg K, Schreier G. Feasibility of a telemedicine framework for collaborative pacemaker follow-up. J Telemed Telecare. 2007;13(7):341-7.
45. Landolina M, Perego GB, Lunati M, Curnis A, Guenzati G, Vicentini A et al. Remote monitoring reduces healthcare use and improves quality of care in heart failure patients with implantable defibrillators: the evolution of management strategies of heart failure patients with implantable defibrillators (EVOLVO) study. Circulation. 2012;125(24):2985-92.
46. Lau CP, Zhang S. Remote monitoring of cardiac implantable devices in the Asia-Pacific. Europace. 2013;15 Suppl 1:i65-i68.
47. Lazarus A. Remote, wireless, ambulatory monitoring of implantable pacemakers, cardioverter defibrillators, and cardiac resynchronization therapy systems: analysis of a worldwide database. Pacing Clin Electrophysiol. 2007;30 Suppl 1:S2-S12.
48. Lee SS, Salole E. Innovative Medical Technology, Health Technology Assessment, and Health Policy: The Case of Remote Patient Monitoring of Cardiac Implantable Electronic Devices in South Korea. Telemed J E Health. 2017;23(1):25-9.
49. Linde C, Abraham WT, Gold MR, St John Sutton M, Ghio S, Daubert C; REVERSE (REsynchronization reVErses Remodeling in Systolic left vEntricular dysfunction) Study Group. Randomized trial of cardiac resynchronization in mildly symptomatic heart failure patients and in asymptomatic patients with left ventricular dysfunction and previous heart failure symptoms. J Am Coll Cardiol. 2008;52(23):1834-43.
50. López-Villegas A, Catalán-Matamoros D, Robles-Musso E, Peiró S. Workload, time and costs of the informal cares in patients with tele-monitoring of pacemakers: the PONIENTE study. Clin Res Cardiol. 2016;105(4):307-13.
51. Louis AA, Turner T, Gretton M, Baksh A, Cleland JG. A systematic review of telemonitoring for the management of heart failure. Eur J Heart Fail. 2003;5:583-90.

REFERENCES NOT INCLUDED IN THE SYSTEMATIC REVIEW

52. Lunati M, Gasparini M, Santini M, Landolina M, Perego GB, Pappone C et al. Follow-up of CRT–ICD: implications for the use of remote follow-up systems. Data from the InSync ICD Italian Registry. Pacing Clin Electrophysiol. 2008;31(1):38-46.
53. Mabo P, Inserm R, Defaye P et al. EVATEL: Remote Follow-up of Patients Implanted with an ICD:the Prospective randomized EVATEL study. ESC Congress report (abstract). European Society of Cardiology Congress 2011.
54. Mabo P, Victor F, Bazin P, Ahres S, Babuty D, Da Costa A et al. A randomized trial of long-term remote monitoring of pacemaker recipients (the COMPAS trial). Eur Heart J. 2012;33(9):1105-11.
55. Maillard N, Perrotton F, Delage E, Gourraud JB, Lande G, Solnon A, Probst V, Grimandi G, Clouet J. Cardiac remote monitoring in France. Arch Cardiovasc Dis. 2014;107(4):253-60.
56. Maisel WH, Stevenson WG, Epstein LM. Changing trends in pacemaker and implantable cardioverter defibrillator generator advisories. Pacing Clin Electrophysiol. 2002;25(12):1670-8.
57. Maisel WH, Hauser RG, Hammill SC, Hauser RG, Ellenbogen KA, Epstein AE et al. Recommendations from the Heart Rhythm Society Task Force on Lead Performance Policies and Guidelines: developed in collaboration with the American College of Cardiology (ACC) and the American Heart Association (AHA). Heart Rhythm. 2009;6(6):869-85.
58. Marinskis G, van Erven L, Bongiorni MG, Lip GY, Pison L, Blomström-Lundqvist C; Scientific Initiative Committee, European Heart Rhythm Association. Practices of cardiac implantable electronic device follow-up: results of the European Heart Rhythm Association survey. Europace. 2012;14(3):423-5.
59. Marzegalli M, Lunati M, Landolina M, Perego GB, Ricci RP, Guenzati G et al. Remote monitoring of CRT–ICD: the multicenter Italian CareLink evaluation— ease of use, acceptance, and organizational implications. Pacing Clin Electrophysiol. 2008;31(10):1259–64.
60. Mascioli G, Curnis A, Landolina M, Klersy C, Gelmini GP, Ruffa F; ATHENS Investigators. Actions elicited during scheduled and unscheduled in-hospital follow-up of cardiac devices: results of the ATHENS multicentre registry. Europace. 2011;13(12):1766-73.
61. Masella C, Zanaboni P, Di Stasi F, Gilardi S, Ponzi P, Valsecchi S. Assessment of a remote monitoring system for implantable cardioverter defibrillators. J Telemed Telecare. 2008;14(6):290-4.
62. Mathur G, Stables RH, Heaven D, Stack Z, Lovegrove A, Ingram A et al. Cardiac pacemaker lead extraction using conventional techniques: a single centre experience. Int J Cardiol. 2003;91(2-3):215-9.
63. Mercader Cuesta J, Rodríguez Barrios JM, Caro JA, Ward J. Impacto económico y sanitario del tratamiento de la bradicardia con marcapasos bicameral vs. monocameral (VVI/R VS. DDD/R). Rev Esp Econ Salud. 2007;6(5):294-302.
64. Mitton CR, Rose MR, Koshman ML, Sheldon RS. Cost-Utility Analysis of Pacemakers for the Treatment of Vasovagal Syncope. Am J Cardiol. 1999;84(11):1356-9.
65. Moss A, Hall W, Cannom D, Klein H, Brown M, Daubert J et al. Cardiac-resynchronization therapy for the prevention of heart-failure events. N Engl J Med. 2009;361(14):1329-38.
66. Neuzil P, Taborsky M, Holy F, Wallbrueck K. Early automatic remote detection of combined lead insulation defect and ICD damage. Europace. 2008;10(5):556–7.
67. Neyt M, Stroobandt S, Obyn C, Camberlin C, Devriese S, De Laet C, Van Brabandt H. Cost-effectiveness of cardiac resynchronisation therapy for patients with moderate-to-severe heart failure: a lifetime Markov model. BMJ Open. 2011;1(2):e000276.
68. Nielsen JC, Kottkamp H, Zabel M, Aliot E, Kreutzer U, Bauer A et al. Automatic home monitoring of implantable cardioverter defibrillators. Europace. 2008;10(6):729-35.
69. Noyes K, Veazie P, Hall WJ, Zhao H, Buttaccio A, Thevenet-Morrison K et al. Cost-effectiveness of cardiac resynchronization therapy in the MADIT-CRT trial. J Cardiovasc Electrophysiol. 2013;24(1):66-74.

REFERENCES NOT INCLUDED IN THE SYSTEMATIC REVIEW

70. O'Brien BJ, Blackhouse G, Goeree R, Healey JS, Roberts RS, Gent M et al. Cost-effectiveness of physiologic pacing: Results of the Canadian Health Economic Assessment of Physiologic Pacing. Heart Rhythm. 2005;2(3):270-5.
71. Ochagavía Calvo A, Baigorri González F. Selección del modo de estimulación del marcapasos. Med Intensiva. 2006;30(5):218-22.
72. Olen MM, Dechert-Crooks B. Implantable cardiac devices: the utility of remote monitoring in a paediatric and CHD population. Cardiol Young. 2017;27(S1):S143-S146.
73. Osca J, Sancho M, Navarro J, Cano O, Raso R, Castro J et al. Fiabilidad técnica y seguridad clínica de un sistema de monitorización remota de dispositivos cardiacos antiarrítmicos. Rev Esp Cardiol. 2009;62(08):886-95.
74. Osman F, Krishnamoorthy S, Nadir A, Mullin P, Morley-Davies A, Creamer J. Safety and cost-effectiveness of same day permanent pacemaker implantation. Am J Cardiol. 2010;106(3):383-5.
75. Ošmera O, Bulava A.The benefits of remote monitoring in long-term care for patients with implantable cardioverter-defibrillators. Neuro Endocrinol Lett. 2014;35(Suppl 1):40-8.
76. Raatikainen M, Uusimaa P, van Ginneken M, Janssen J, Linnaluoto M. Remote monitoring of implantable cardioverter defibrillator patients: a safe, time-saving, and cost-effective means for follow-up. Europace. 2008;10(10):1145-51.
77. Ren X, Apostolakos C, Vo TH, Shaw RE, Shields K, Banki NM et al. Remote monitoring of implantable pacemakers: in-office setup significantly improves successful data transmission. Clin Cardiol. 2013;36(10):634-7.
78. Ribeiro AL, Rincón LG, Oliveira BG, Mota CC, Pires MT. Enhancing longevity of pacemakers through reprogramming. Underutilization and cost-effectiveness. Arq Bras Cardiol. 2001;76(6):437-44.
79. Ricci RP, Morichelli L, Santini M. Home monitoring remote control of pacemaker and implantable cardioverter defibrillator patients in clinical practice: impact on medical management and health-care resource utilization. Europace. 2008;10(2):164-70.
80. Ricci RP, Morichelli L, Santini M. Remote control of implanted devices through Home Monitoring technology improves detection and clinical management of atrial fibrillation. Europace. 2009;11(1):54-61.
81. Ricci RP, Morichelli L, Quarta L, Sassi A, Porfili A, Laudadio MT et al. Long-term patient acceptance of and satisfaction with implanted device remote monitoring. Europace. 2010;12(5):674-9.
82. Ricci RP, D'Onofrio A, Padeletti L, Sagone A, Vicentini A, Vicentini A et al. Rationale and design of the health economics evaluation registry for remote follow-up: TARIFF. Europace. 2012;14(11):1661-5.
83. Ricci RP, Morichelli L, D'Onofrio A, Calò L, Vaccari D, Zanotto G et al. Effectiveness of remote monitoring of CIEDs in detection and treatment of clinical and device-related cardiovascular events in daily practice: the HomeGuide Registry. Europace. 2013;15(7):970-7.
84. Ricci RP, Locati ET, Campana A, Cavallaro C, Giammaria M, Landolina M et al. Monitoraggio remoto dei dispositivi cardiaci impiantabili: health technology assessment [Remote monitoring of implantable cardiac devices: health technology assessment]. G Ital Cardiol (Rome). 2015;16(5):295-303. Italian.
85. Ricci RP, Vicentini A, D'Onofrio A, Sagone A, Rovaris G, Padeletti L et al. Economic analysis of remote monitoring of cardiac implantable electronic devices: Results of the Health Economics Evaluation Registry for Remote Follow-up (TARIFF) study. Heart Rhythm. 2017;14(1):50-57.
86. Roda J, Rodríguez Barrios JM, Caro I, Deniz HB, Xenakis JJ, Ward A et al. Modelización del beneficio clínico y del impacto económico del implante de marcapasos con modo de estimulación ventricular minimizada. Arch Cardiovasc Dis. 2011;104(5):332-42.
87. Rohde LE, Zimerman LI, Pimentel M, Polanczyk CA. Cost-effectiveness of cardiac resynchronization therapy in patients with heart failure: The perspective of a middle-income country's public health system. Int J Cardiol. 2013;163(3):309-315.

REFERENCES NOT INCLUDED IN THE SYSTEMATIC REVIEW

88. Sakurai T, Tokutsu S, Nishimura O, Tagami Y, Takimoto M, Ohta H et al. Surveillance and follow-up of patients with implanted cardiac pacemaker by telephone transmission. Jpn Heart J. 1978;19(6):829-38.
89. Santini M, Ricci RP, Lunati M, Landolina M, Perego GB, Marzegalli M et al. Remote monitoring of patients with biventricular defibrillators through the CareLink system improves clinical management of arrhythmias and heart failure episodes. J Interv Card Electrophysiol. 2009;24(1):53–61
90. Schoenfeld SH, Compton SJ, Mead RH, Weiss DN, Sherfesee L, Englund J et al. Remote monitoring of implantable cardioverter defibrillators: a prospective analysis. Pacing Clin Electrophysiol. 2004;27 (6 Pt 1):757-63.
91. Sloan FA, George LK, Hu L. Emergency room and inpatient use after cardiac pacemaker implantation. Am J Cardiol. 2013;111(4):563-8.
92. Slotwiner D, Wilkoff B. Cost efficiency and reimbursement of remote monitoring: a US perspective. Europace. 2013;15 Suppl 1:i54-i58.
93. Spencker S, Coban N, Koch L, Schirdewan A, Muller D. Potential role of home monitoring to reduce inappropriate shocks in implantable cardioverter defibrillator patients due to lead failure. Europace. 2009;11(4):483-8.
94. Stevenson RT, Lugg D, Gray R, Hollis D, Stoner M, Williams JL. Pacemaker implantation in the extreme elderly. J Interv Card Electrophysiol 2012;33(1):51-8.
95. Stoepel C, Boland J, Busca R, Saal G, Oliveira M. Usefulness of remote monitoring in cardiac implantable device follow-up. Telemed J E Health 2009;15(10):1026-30.
96. Sweesy MW, Erickson SL, Crago JA, Castor KN, Batey RL, Forney RC. Analysis of the effectiveness of in-office and transtelephonic follow-up in terms of pacemaker system complications. Pacing Clin Electrophysiol. 1994;17(11 Pt 2):2001-3.
97. Tarride J, Morgan L, DesMeules M, Luo W, Burke N, O´Reilly D. A review of the cost of cardiovascular disease. Can J Cardiol. 2009;25(6):e195-202.
98. Thokala P, Baalbaki H, Brennan A, Pandor A, Stevens JW, Gomersall T et al. Telemonitoring after discharge from hospital with heart failure: cost-effectiveness modelling of alternative service designs. BMJ Open 2013;3(9):e003250.
99. Tuppin P, Neumann A, Marijon E, de Peretti C, Weill A, Ricordeau P et al. Implantation and patient profiles for pacemakers and cardioverter-defibrillators in France (2008—2009). Arch Cardiovasc Dis. 2011;104(5):332-42.
100. Udo EO, Zuithoff NP, van Hemel NM, de Cock CC, Hendriks T, Doevendans PA. Incidence and predictors of short- and long-term complications in pacemaker therapy: the FOLLOWPACE study. Heart Rhythm. 2012;9(5):728-35.
101. van Eck JW, van Hemel NM, van den Bos A, Taks W, Grobbee DE, Moons KG. Predictors of improved quality of life 1 year after pacemaker implantation. Am Heart J. 2008;156(3):491-7.
102. van Eck JW, van Hemel NM, Zuithof P, van Asseldonk JP, Voskuil TL, Grobbee DE et al. Incidence and predictors of in-hospital events after first implantation of pacemakers. Europace. 2007;9(10):884-9.
103. Varma N, Stambler B, Chun S. Detection of atrial fibrillation by implanted devices with wireless data transmission capability. Pacing Clin Electrophysiol. 2005;28(Suppl 1):S133-6.
104. Varma N, Epstein AE, Irimpen A, Schweikert R, Love C; TRUST Investigators. Efficacy and safety of automatic remote monitoring for implantable cardioverter-defibrillator follow-up: the Lumos-T Safely Reduces Routine Office Device Follow-up (TRUST) trial. Circulation. 2010;122(4):325-32.
105. Varma N, Michalski J, Epstein AE, Schweikert R. Automatic remote monitoring of implantable cardioverter-defibrillator lead and generator performance: the Lumos-T Safely RedUceS RouTine Office Device Follow-Up (TRUST) trial. Circ Arrhythm Electrophysiol. 2010;3(5):428-36.
106. Varma N, Ricci RP. Telemedicine and cardiac implants: what is the benefit? Eur Heart J. 2013;34(25):1885-95.
107. Vogtmann T, Stiller S, Marek A, Kespohl S, Gomer M, Kühlkamp V et al. Workload and usefulness of daily centralized Home Monitoring for patients treated with implantable

cardiac pacing devices: Results of the MoniC (Model Project Monitor Centre) prospective multicentre study. Europace 2013;15(2):219–26.

108. Wiegand UK, Potratz J, Bode F, Schreiber R, Bonnemeier H, Peters W et al. Cost-effectiveness of dual-chamber pacemaker therapy: does single lead VDD pacing reduce treatment costs of atrioventricular block?. Eur Heart J. 2001;22(2):174-80.
109. Wilkoff BL, Auricchio A, Brugada J, Cowie M, Ellenbogen KA, Gillis AM, et al. HRS/EHRA expert consensus on the monitoring of cardiovascular implantable electronic devices (CIEDs): description of techniques, indications, personnel, frequency and ethical considerations. Heart Rhythm 2008;5(6):907–925.
110. Wu J, Kessler DK, Chakko S, Kessler KM. A cost-effectiveness strategy for transtelephonic arrhythmia monitoring. Am J Cardiol. 1995;75(2):184-5.
111. Zabel M, Müller-Riemenschneider F, Christoph Geller FJ, Brachmann J, Kühlkamp V, Dissmann R et al. Rationale and design of the MONITOR-ICD study: A randomized comparison of economic and clinical effects of automatic remote MONITORing versus control in patients with Implantable Cardioverter Defibrillators. Am Heart J.2014;168(4):430-7.
112. Zanaboni P, Landolina M, Marzegalli M, Lunati M, Perego GB, Guenzati G et al. Cost-utility analysis of the EVOLVO study on remote monitoring for heart failure patients with implantable defibrillators: randomized controlled trial. J Med Internet Res. 2013;15(5):e106.
113. Zhan C, Baine WB, Sedrakyan A, Steiner C. Cardiac device implantation in the United States from 1997 through 2004: a population-based analysis. J Gen Intern Med. 2008;23(Suppl 1):13-9.
